# A Review of Environmental Context Detection for Navigation Based on Multiple Sensors

**DOI:** 10.3390/s20164532

**Published:** 2020-08-13

**Authors:** Florent Feriol, Damien Vivet, Yoko Watanabe

**Affiliations:** 1 Optronics and Signal Research Group, ISAE-SUPAERO, 31055 Toulouse, France; damien.vivet@isae-supaero.fr; 2 Department of Information Processing and Systems, ONERA, 31055 Toulouse, France; yoko.watanabe@onera.fr

**Keywords:** context detection, environmental context detection, context-aware navigation, UGV, unmanned ground vehicle, vision, image processing, GNSS signal, sky extraction, scene analysis, aerial photography segmentation, remote sensing, signal processing

## Abstract

Current navigation systems use multi-sensor data to improve the localization accuracy, but often without certitude on the quality of those measurements in certain situations. The context detection will enable us to build an adaptive navigation system to improve the precision and the robustness of its localization solution by anticipating possible degradation in sensor signal quality (GNSS in urban canyons for instance or camera-based navigation in a non-textured environment). That is why context detection is considered the future of navigation systems. Thus, it is important firstly to define this concept of context for navigation and to find a way to extract it from available information. This paper overviews existing GNSS and on-board vision-based solutions of environmental context detection. This review shows that most of the state-of-the art research works focus on only one type of data. It confirms that the main perspective of this problem is to combine different indicators from multiple sensors.

## 1. Introduction

In the past decade, much work has been done to make navigation more precise and more reliable: sensor fusion, improvement of signal quality, receiver hardware enhancement (antennas arrays for instance), mitigation of errors, robust filtering, and so on. Fusing data from different sensors is a good way to make a navigation system more accurate, but it could also degrade the navigation accuracy when some of those data are erroneous. For instance, fusing Global Navigation Satellite System (GNSS) data with vision-based data in an indoor situation or in a harsh environment would create positioning errors since GNSS data might be unreliable in such a context. That is why context information is interesting and useful for navigation purposes, as mentioned in [1,2]. There are two very different concepts of "context" for navigation: behavioural and environmental contexts. The behavioural context represents the activity of either a vehicle in the case of robotic applications such as Unmanned Ground/Aerial Vehicles (UGVs or UAVs) (for example, accelerating/slowing down) or a user in the case of a smartphone (climbing stairs, running, taking a lift, etc.). The environmental context defines the surroundings, like indoor/outdoor, close to a building, under a tree, and so on. Reference [1] is an interesting introduction to environmental contexts since it gives an exhausting list including even the space or submarine environment.

**Figure 1:**
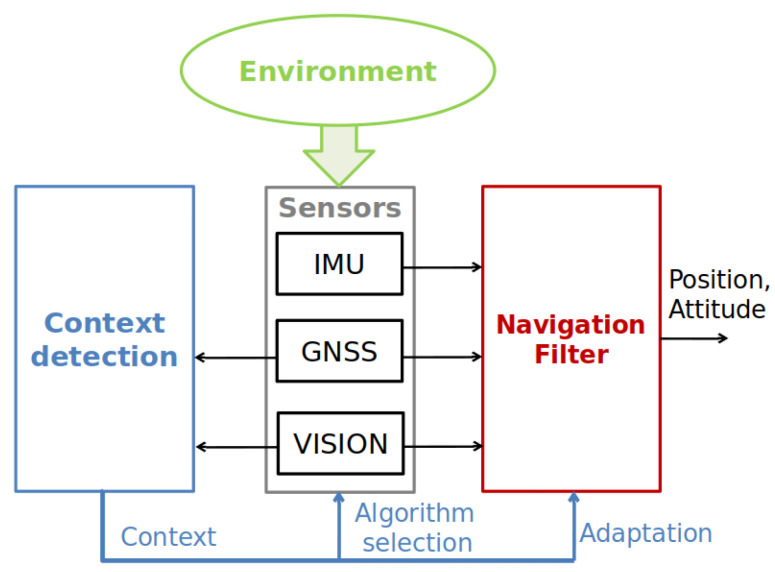
Context-aware adaptive navigation concept.

Context-aware navigation is a hot topic for pedestrian applications where human behaviour is detected and classified mainly using Inertial Measurement Unit (IMU) sensor data available in smartphones [3,4,5] (see also Section 4). However, when limiting our scope to on-/near-ground vehicle navigation, the behavioural context is of less interest (even if, in recent years, some papers have shown that it is possible to use the behavioural context to help environmental context detection [3,6,7]). Instead, as illustrated in the previous example of GNSS, the environmental context can give important information about the potential quality of certain navigation sensor signals. In the literature today, environmental context awareness is mostly used in GNSS applications for satellite mitigation, but not in a more general framework of navigation. In addition, few works focus on multi-sensor fusion for context detection. Therefore, the objective of this paper is to explore the multi-sensor solutions to robustly classify the environmental contexts that have an impact on the sensor quality. Such context detection could be used to adapt data fusion in cases where one (or more) of the sensor data is possibly degraded (e.g., GNSS in indoor situations, a camera in a non-textured environment). It could also help to choose a proper data processing algorithm considering the expected sensor signal or data quality in a particular context. This paper assumes a classical onboard sensor set for ground vehicle (or drone) navigation: GNSS receiver, IMU, and camera(s). Indeed, adding extra sensors would augment the context detection capability, but on the other hand, it is very important to maintain the viability of the system. For this reason, this paper primarily focuses on a review of environmental context detection methods based on these most frequently used navigation sensors. Nonetheless, we will give a glance at methods based on other types of sensors in Section 4. Figure 1 illustrates a scheme of the context-aware adaptive navigation system with the considered sensor set, and Table 1 lists the environmental contexts along with their impacts on the GNSS and vision sensors’ quality. As shown in Figure 1, adaptation could be applied both to data extraction in each sensor system and to data fusion in the navigation filter (a list of different solutions is available in Table 1).

Indeed, some algorithms for data extraction are designed to be more successful under particular conditions. For instance, algorithms used to extract navigation information from camera images can be selected depending on the context. In indoor or urban environments, which mainly contain structured buildings and handmade objects, algorithms based on line detection as a key feature could be more appropriate and efficient. In outdoor environments such as natural areas, algorithms should rather be based on point feature detection, as explained in [35].

**Figure 2:**
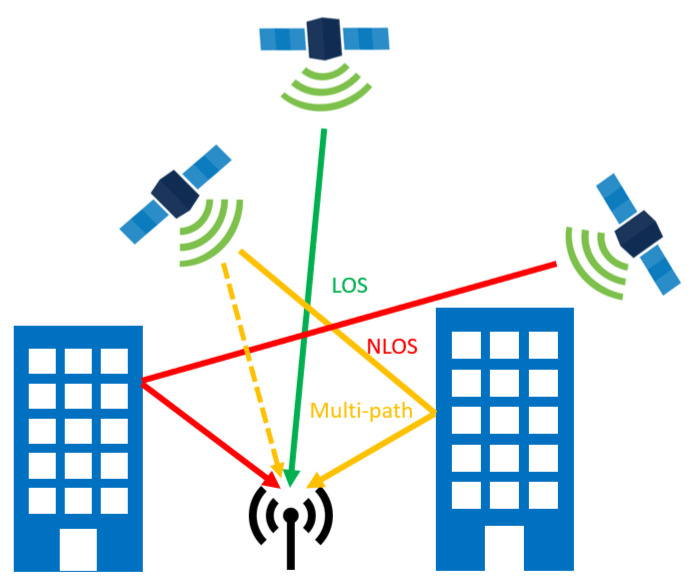
Schemes of the multipath and NLOS effect.

The quality of the GNSS signals also significantly depends on the environment configuration. The GNSS signals can be attenuated in urban environments, but also under trees, as shown in [36,37,38]. In such conditions where the signal is weak, the coherent integration time duration can be extended to improve the signal detection [39]. Another known problem of GNSS in harsh environment is the potential signal reflections on surfaces. This changes the travel distance from the satellite to the receiver. This effect creates an error in the position solution calculated by the receiver. Two different scenarios exist: multipaths and None Line Of Sight (NLOS). Examples of multipath and NLOS situations are shown in Figure 2. In NLOS scenarios, one (or more) GNSS signal is masked in the direct path, but reaches the receiver via a reflected path. Most of the time, the signal blocking element is a tall structure (e.g., a building), which means that NLOS exists primarily in urban areas. Since NLOS creates higher errors of positioning than the multipath effect [40], it is important to find a way to mitigate this problem. One solution is to modify and adapt the way GNSS data are fused with the other sensors in the navigation filter. The tight-coupling, which uses the pseudoranges and the Doppler estimations, has the advantage of needing fewer available satellites (down to two), which is very beneficial in cluttered environments such as urban canyons where many satellites can be NLOS. On the contrary, the loose-coupling is simpler and thus is more efficient in terms of computational load, but performs poorer in harsh environments where the position cannot be easily computed with pseudoranges. It is also more robust to GNSS and INS faults since one cannot impact the other. Another solution is the NLOS satellites’ filtering thanks to image processing, as explained in Section 3.1, which can also be useful to limit the near-far effect [34]. The multipath effect scenario happens when there is a combination of the direct and reflected path signals (Figure 2). Reference [41] explained that there are three different types of multipath effects:

- Diffuse multipath typically happens when the signal encounters a cluttered metallic surface such as overhead wires. The signal is diffused in a wide variety of directions, creating an error of positioning, which can go up to 10 m.
- Specular multipath appears with reflective surfaces such as a mirror and glass and can lead to positioning errors between two and six m.
- Water reflections are linked to the presence of a water surface next to the antenna and can create positioning errors of an order of 10 m.

Multipath mitigation can be done thanks to maximum-likelihood methods such as MMT [42] (Multipath Mitigation Technology), MEDLL [43] (Multipath Estimation Delay Lock Loop), and FIMLA [44] (Fast Iterative Maximum Likelihood Algorithm), but require much computational power. It is also possible to use Doppler measurements as additional information since multipath has a lower impact on them than on pseudoranges [45,46]. Doppler methods can also be used in situations where the GNSS signal is very noisy (under a canopy, semi-indoor areas, etc.). Other GNSS limitations that are not really linked to a particular environmental context, such as jamming/spoofing, will not be considered in this paper.

If there is a means to detect the environment context correctly, those aforementioned adaptation schemes can be applied to the navigation system. Such context-aware adaptive navigation solutions will improve the navigation performance in terms of both the accuracy and the reliability, which can help current navigation applications or even lead to new ones. As presented later in this review, most of the existing context-aware solutions base their context detection only on one type of indicator from one sensor (GNSS or vision) and are basically designed to do sensor management in the binary indoor/outdoor situation. Unlike these, our research interest and perspective is to propose new multi-indicator/multi-sensor-based solutions that are capable of detecting and classifying the environmental contexts more finely and robustly for the navigation adaptation purpose.

The paper is organized as follows: Section 2 presents different context indicators derived from the GNSS signals. Section 3 focuses on context detection by vision sensors. Section 4 briefly introduces techniques that are based on others sensors. Finally, Section 5 provides the summary and our perspectives towards context detection for navigation.

## 2. GNSS Signal-Based Context Indicators

It is natural to think of using the characteristics of the GNSS signal itself to detect the context affecting it. Different indicators can be used for this purpose and are presented in the following parts.

### 2.1. C/N0

The Carrier to Noise ratio (C/N0) is probably the most well-known indicator of the GNSS signal. It is a signal-to-noise ratio that is not quantified in dB, but in dB.Hz. It gives information on the signal quality and on how badly (or not) the signal has been attenuated. According to [34], a standard C/N0 is around 45 dB.Hz in an open sky area. They also showed that different ranges of C/N0 values can be representative of a specific context (Table 2).

When looking at this table, it seems very easy to distinguish indoor environments from outdoor ones: a simple threshold seems more than enough. However, the reality is much more complex. Firstly, C/N0 is not that stable and reliable. This was showcased in [1], where they acquired 100 s of C/N0 values (at a frequency of 1Hz) in different contexts such as indoors, outdoors in urban/residential areas, and open field. For each of those recordings, the authors computed histograms of C/N0. Those histograms showed that C/N0 values vary greatly during the period of acquisition (due to temporal losses of satellite visibility for a short time period). As a conclusion, we can fairly say that it is not possible to implement a simple threshold, as given in Table 2, to detect a context. However, the authors of [1] made two observations based on those data:

- The average C/N0 value is higher in outdoor environments than in indoor ones.
- The standard deviation is larger in outdoor environments than in indoor ones, which makes sense since signal occlusion is more likely to happen in outdoor environments because a small change in the satellite constellation can greatly affect the satellite visibility in a cluttered environment.

Those indicators of C/N0 (average and variance) seem useful to make a classification of the indoor/outdoor context. The problem is that we need an integration time long enough to obtain reliable values for these indicators. When considering the vehicle navigation application, the GNSS receiver is not static and travels in the environment during the data sampling time window. This displacement can create the unreliability of C/N0 values. Secondly, the definitions of the soft-indoor and intermediate context are not very clear. A GNSS receiver can be for instance placed at the exit of a building and therefore should be considered as intermediate, but the same classification can happen with a receiver close to a window, which should be classified as soft-indoor. This means that GNSS indicators cannot classify context robustly in certain situations where the performances are variable.

The problem of movement was addressed in [19] where they built a setup with the receiver antenna on the back of a human who was moving between indoor and outdoor locations. In the recorded data, there was a clear difference of the C/N0 values when passing from outdoors to indoors. There was a diminution of the average value, as well as an increase in noise (higher variance). This noise makes it difficult to detect a transition between outdoors and indoors with a simple threshold. The authors of [19] proposed a solution based on a Rice law (probability distribution), which is described by a factor *K*. The goal is to estimate the value of *K*, which corresponds to the fading of the signal and is in dB. More details on how to estimate the *K*-Rician factor can be found in [47]. Thanks to the *K*-Rician factor, most of the noise is removed and the signal becomes much more stable. It is then possible to classify rather easily the indoor and outdoor contexts. To achieve this classification, two thresholds were used in the paper. If C/N0 < 32 dB-Hz and K< 10 dB, then the context was declared as indoor. The authors also tried different sampling times: 100 ms, 500 ms, and 1 s. At the end, the sampling time of one second gave the best result, which was not very surprising considering the amount of noise on the 100 ms signal. In [48], they also showed that the Rician-factor method works for various velocities (obviously, the higher the velocity is, the higher the variations will be). However, this indoor/outdoor classification based on the *K*-Rician factor and C/N0 values was only used as an indicator to switch between processing strategies (vector-based and block-based), and the article did not mention the accuracy of the context classification, but only of the navigation solution as a whole.

Indoor/outdoor classification by the C/N0 indicator seems to perform well thanks to the *K*-Rician factor, but the article [20] went even beyond that and performed the context classification into indoors, outdoors, and urban. They used a bottom-up strategy where they firstly tried to classify indoors, hybrid, and outdoors. To do so, they implemented an HMM (Hidden Markov Model) with two features:

- The number of satellites with a C/N0 > 25 dB-Hz. This idea of the number of visible satellites was also exploited in [21] (SatProbe) to classify indoors from outdoors (only based on the GPS constellation).
- The sum of the C/N0 of satellites > 25 dB-Hz

Once the first classification was done and if the detected context was outdoors, a fuzzy inference system with the following two features was used to find whether the receiver was in an open sky or in an urban environment:

- The sum of squares of the pseudo-range residuals, which is defined as follows:
  (1) $$zPRR=(\sum_{i=1}^{N}{\left|\rho_{c}-\rho_{e}\right|}^{2})/\left(N-4\right)$$
  with *N* the number of satellites, $\rho_{c}$ the measured pseudo-range, and $\rho_{e}$ the estimated range.
- The sum of the C/N0 values of satellites >25 dB-Hz

The overall system had a success rate of 88.2%, which was a promising result. The limitation of this method is that the most challenging context in terms of classification was only described as intermediate. This means that we do not really know the nature of those intermediate contexts, and it leaves us with a big problematic: Can we still use GNSS reliably in those contexts? The authors also only used a unique constellation to build their indicators, and a multi-constellation solution could enhance the results. Another problem, which was not addressed in the previous papers, is the robustness of those methods since GNSS signal quality depends on the location and type receiver, its displacement velocity, atmospheric conditions/weather, and even time (since it has an influence on the constellation). As a consequence, the threshold values provided cannot be trusted in all scenarios.

### 2.2. Pseudo-Range

Another indicator that is used to classify LOS signals from NLOS signals is the pseudo-range residuals, as proposed in [49]. As defined in Section 2.1, the pseudo-range residuals are the difference between the measured pseudo-range (thanks to the satellite ephemeris data and the signal travelling time) and the estimated pseudo-range that is obtained once the user position has been computed. Being able to distinguish LOS from NLOS is interesting for the context detection since a large number of NLOS satellites means that the context is indoors or at least dense urban. The principle is simple: if a satellite is in an NLOS situation, its measured pseudo-range should be longer than the estimated one due to a longer signal path. One problem noticed in [49] is that pseudo-range residuals are biased due to the fact that the estimated user position is calculated from the measured pseudo-ranges. At least four satellites are needed in order to obtain a position solution, and some of them can be NLOS. Thus, the position admits some errors. To reduce this bias effect, the authors of [49] proposed to use the second derivative of the pseudo-range residuals. Variations of this indicator are much larger in the case of NLOS than in LOS and hence could help us to define if we are in an urban environment. However, the main issue is that we need a certain integration time to be sure to observe some important variations. Although this indicator cannot work alone, it can be used in some uncertain detection cases where additional data are needed to confirm the context. It is also noteworthy that the authors tried to use the Doppler shift to classify LOS from NLOS. Unfortunately, this solution does not really work in low or null velocity situations since the Doppler shift represents the relative speed between the receiver and the satellite.

### 2.3. Satellite Elevations

Satellite elevation is not an indicator that gives directly any information on the context. However, it can be used as supplementary information to help the context detection, like the pseudo-range. Indeed, the lower the satellite is, the higher the risk is to have its signal occluded by a building. Elevation can be used for detecting multipaths or for NLOS/LOS classification. To do so, it is mandatory to have a height map of the environment to compare buildings height with satellites elevations (azimuths are also used in this process). Formerly, such height maps needed to be created by various methods [50], but currently, 3D city models are widely available [51,52,53,54]. All those techniques do work, they require a 3D environment model and an accurate estimation of the receiver position, which are far from being available everywhere. It was also stated in [55] that those techniques require much computational resource, which cannot be supported by every navigation setup.

Being able to distinguish which signal can be blocked from a 3D map is useful for shadow matching. This technique improves the position precision solution by verifying which signals are receivable in the direct path to reduce the search space of the user position. It is useful especially in very cluttered areas such as urban canyons [56,57,58]. Indeed, if a direct satellite signal is supposed to be LOS, whereas the signal quality coming from that satellite is poor (sometimes, there is no signal at all), this means that the position solution calculated is incorrect and, thus, needs to be recalculated. A new candidate user position close to the initial one is computed by using areas where the satellite is supposed to be blocked. This process can be refined by using other satellites (to be very accurate, this method needs a large number of satellites and is, thus, more efficient with multiple constellations). Shadow matching can be very effective to reduce cross-street error (if the LOS/NLOS classification algorithm is reliable), but is ineffective in the case of along-street errors.

### 2.4. Auto-Correlation Function

The Auto-Correlation Function (ACF) is a bit different from the other GNSS indicators presented so far. While they are all available at the output of the receiver, ACF is not accessible as an output and is only available inside the receiver.

**Figure 3:**
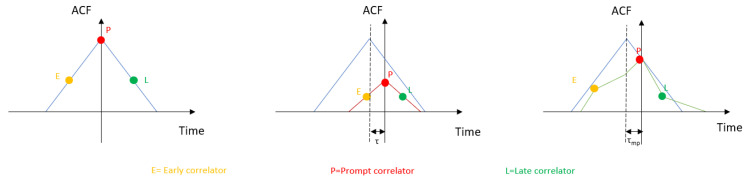
Different shapes of the ACF in LOS (**left**), NLOS (**middle**), and multipath situations (**right**).

ACF is a convolution between the non-distorted local signal replica generated by the receiver and the potentially distorted signal that comes from the satellite [59]. If the two signals are in-phase, which means that both signals are aligned and that the original one is not distorted, the ACF will have a triangular shape with a perfect symmetry between the positive and negative slope. If the satellite signal is distorted, then this triangular shape will be deformed. A good explanation of how the ACF evolves in the case of LOS and NLOS multipath can be seen in Figure 3. The ACF is based on three correlators (early, prompt, and late), which will slightly change the phase when the replica needs to be correlated with the satellite signal. From the example of Figure 3, we can see different patterns:

- In the case of NLOS (middle of Figure 3):
  - Attenuation of the ACF signal
  - Delay in the maximum of correlation (prompt)
- In the case of multipath (right of Figure 3):
  - Attenuation or augmentation of the ACF amplitude
  - Suppression of the symmetry between the positive and negative slope

López-Salcedo et al. [8] proposed to use this slope asymmetry to detect multipath. They begin with the normalization of every function by an estimation of C/N0 in order to be invariant to noise/attenuation and synchronize each sample. Next, the slopes are estimated by the least squares on the three points that are the closest to the maximum of correlation. Then, the values of the two slopes are summed up to create a Slope AsyMmetry indicator (SAM). The SAM value becomes large in urban canyons where multipath occurs, while it stays around zero in the open sky situation. Since the goal of this paper was only to described the GPS signal in harsh environments, we have no information about the success rate of this method. However, an extension of this work showed that the technique was functional for real-time application [60].

Other ACF-based techniques were proposed. Gang et al. [61] used the wavelet transform modulus maxima to detect an “abnormal” ACF shape. The solution was only tested on data coming from the simulation and not tried on real data. Mubarak et al. [14] proposed the ELP (Early Late Phase) indicator, which is the phase difference between the outputs of the early and late correlators. This value becomes high in the presence of multipath and is a good discriminator. This solution was only tested with simulation data. Mubarak et al. [62] showed the compatibility of the method with BOC (Binary Offset Carrier) and BPSK (Binary Phase-Shift Keying) modulation.

Even if detecting multipath by these techniques can be a good insight to get the context, this is not ideal since they are not able to distinguish NLOS from multipath. We can only say that the vehicle is in an environment where GNSS admits some error, but cannot give a precise context. For instance, in the case where there is a majority of NLOS satellite signals, the vehicle is probably in an urban area, and the context is not likely to change soon. On the other hand, if the majority of satellites have the multipath effect (which can be due to some particular materials close to the receiver, buildings, water, and so on), even if the signal quality is not the best, the GNSS can still give a decent position solution. It is then clear that we cannot properly adapt the data fusion if we base the system only on multipath detection.

Skournetou and Lohan [22] introduced an indicator for indoor/outdoor classification called the Level Crossing Rate (LCR), which basically counts the number of times the ACF crosses a threshold (denoted *a*) both from below and above. It is mathematically defined as follows:

(2) $$LCR\left(a\right)=\mathrm{card}\{i|\left({\bar{R}}_{i}\leq a\wedge {\bar{R}}_{i+1}>a\right)\;\;⋀\;\;\left({\bar{R}}_{i+1}\leq a\wedge {\bar{R}}_{i}>a\right)\}$$

$R_{i}$ is the non-coherent average ACF at timestamp defined as:

(3) $$\bar{R}\left(\hat{\tau },{\hat{f}}_{D}\right)=\frac{1}{N_{nc}}\sum_{N_{nc}}{\left|\frac{1}{N_{c}}\sum_{m=1}^{N_{c}}R\left(\hat{\tau },{\hat{f}}_{D},m\right)\right|}^{2}$$

$N_{nc}$ is the number of non-coherent integrations (meaning that the phase is not used during the correlation step since the correlation signal is squared up), while $N_{c}$ is the number of coherent integrations (meaning that the phase is used during the correlation step since the original correlation signal is used). ${\hat{f}}_{D}$ is the Doppler frequency. $\hat{\tau }$ is the delay, and *m* an epoch. Then, a new indicator called the averaged ACF level of max LCR, which actually corresponds to the averaged ACF level where the LCR is at its highest value, is computed. This indicator will be the threshold for discriminating outdoors from indoors. The threshold value was computed after various simulations with C/N0 around 20 dB.Hz (which corresponds to an intermediate environment between indoors and outdoors). They found a threshold for different values of $N_{nc}$ and $N_{c}$. Then, if the value computed for the upcoming signal is lower than the averaged threshold, the receiver is indoors, otherwise it is outdoors. The performance of this detection system varied greatly, between a 67% and 95% successful classification rate depending on the movement of the receiver and the number of multipaths signals. All the tests were conducted in simulation.

### 2.5. Combination of Multiple Indicators

For now, except the ones in [19,20], all the GNSS-based context detection solutions presented so far were based on a single indicator. This part presents techniques that combine several indicators for context detection. Most of them are based on Machine Learning (ML) approaches.

For LOS/NLOS classification, Yozevitch et al. [49] built a binary decision tree thanks to the RapidMiner software. Their tree was based on C/N0, pseudo-range residuals, and satellite elevations. The learning phase was done by using a labelled database created with an Android application where the user can take a picture and compare the satellite elevation with the building boundaries. They achieved a success rate of around 80%. Their results were compared to those of an expectation maximization algorithm, and a similar performance was achieved.

Hsu [55] proposed a method to classify the GNSS signals into three categories: NLOS, multipath, and LOS (clean signal). This is interesting because the number of NLOS satellites could help us to extract some context information like indoors, urban, or open sky. The authors, based in Hong Kong, set up an antenna in a dense building location during 24 h. They labelled the recorded GNSS data between NLOS or LOS by applying the ray tracing method using a 3D terrain model. They used those data as the training population for the SVM (Support Vector Machine) classifier with the following four different features:

- Signal strength (C/N0 value)
- Change rate of the received signal strength
- Pseudo-range residuals
- Difference between delta pseudo-range (5) and pseudo-range rate (6), defined as follows (4):
  (4) $$|\Delta \rho -\dot{\rho }\cdot \Delta t|$$
  where Δρ is the difference between the actual and previous pseudo-range measurements:
  (5) $$\Delta \rho_{k}^{\left(i\right)}=\rho_{k}^{\left(i\right)}-\rho_{k-1}^{\left(i\right)}$$
  and $\dot{\rho }$ is the difference in Doppler shift ($f_{\mathrm{Doppler}}$ is the Doppler shift, *c* the celerity, and $f_{L1}$ the GPS L1 band carrier frequency):
  (6) $${\dot{\rho }}^{\left(i\right)}=f_{\mathrm{Doppler}}^{\left(i\right)}-\frac{c}{f_{L1}}$$

With a population of 85,365 samples and using an SVM classifier on the signal strength and the difference between delta pseudo-range and pseudo-range rate, they achieved a 75% correct classification. Using all four features did not improve the classification performance, so we can fairly say that the features used here are not the best set.

Xu et al. [63] also tried to classify GNSS signals into three different categories, LOS, NLOS, and multipath, using to an SVM model. They compared three different classifiers. The first one, called NMEA (National Marine Electronics Association), is based on three different GNSS features: C/N0 value, satellites’ elevations, and the difference between delta pseudo-range and pseudo-range rate. The second classifier, RINEX (Receiver Independent Exchange Format), is based only on C/N0 and satellites’ elevations. The last classifier called “correlator” is based on four features extracted from the ACF: the ratio between the maximum correlation value measured and the standard value (open sky area), the mean and variance of the correlation peak delay, and ELP. The labelling of the training population was done by using a 3D model but with the sky mask and not ray tracing. However, the sky mask does not work very well with satellites whose line-of-sight passes close to the edges of a building and does not detect multipath. The authors proposed to use the double difference of the carrier phase to help the labelling. They trained their RINEX and NMEA model with 6802 samples and their correlator model with 69,000 samples. The final results can be found in Table 3. We can conclude that the classifier on the ACF function works better than the other two. This is quite logical since the ACF gives more information that is far more robust than C/N0 values, which have a large variance. However, we have the same observation as in Section 2.4: accessing the ACF costs much and is not granted for every receiver.

In [9], the authors tried to detect transitions between indoor and outdoor contexts. To do so, four different families were created: deep indoors (no window, no balcony), shallow indoors (the opposite of deep indoors), semi outdoors (outdoors, but many buildings are surrounding the user position), and open outdoors (clear sky). However, the authors of [9] finally decided to only distinguish indoors from outdoors, so the final classification was a simple binary case. In order to classify the signals, they started by selecting 36 different features, which belonged to three categories:

- Spatial geometry
  - Azimuth distribution of the satellite
  - Satellite azimuth distribution proportion
  - Proportion of the number of satellites within a range of 90${}^{\circ }$ of the azimuth
  - Proportion of the number of satellites within a range of 180${}^{\circ }$ of the azimuth
  - Position Dilution of Precision (PDoP), Vertical Dilution of Precision (VDoP), Horizontal Dilution of Precision (HDoP)
- Time sequence
  - The number of visible satellites from time t_2_ to time t_1_
  - The ratio of satellites, the CNR (Carrier-to-Noise Ratio) of which decreases from time t_2_ to time t_1_
  - The ratio of satellites, the CNR of which holds from time t_2_ to time t_1_
  - The ratio of satellites, the CNR of which decreases from time t_2_ to time t_1_
- Statistical
  - Number of satellites at the current time
  - Mean, variance, standard deviation, minimum, maximum, median, range, interquartile range, skewness, kurtosis of all visible satellites’ CNR
  - Mean, variance, standard deviation, minimum, maximum, median, range, interquartile range, skewness, kurtosis of visible satellites’ CNR under different sliding window lengths
  - Mean of PDoP, VDoP, and HDoP

Different ML algorithms were applied on those 36 features: RF (Random Forest), SVM, AdaBoost (or Adaptive Boosting), XGBoost, and LightGBM (Gradient Boosting Machine). Another algorithm called "stacking" was also tried. This algorithm combines all the outputs of the previous ML algorithms except SVM to train a new layer called the meta-classifier. Finally, they tried a last classifier based on the stacking one, but with an HMM as the final layer. Four different datasets, which represented more than 195,851 samples, were created for their classification test. Those datasets were acquired at different locations, with different scenarios and sensors. The best classification results were obtained for almost every test by the last classifier, the Stacking algorithm with the HMM model. Using all three types of features increased the success rate of classification, but by a very small margin. Using only spatial or temporal features led to a similar result. LightGBM was the most efficient single model in terms of classification. The limit of this article was that they only focused on indoor/outdoor classification and not on a more precise environmental context (urban, open-field, etc.).

Overall, the main drawback of supervised ML is that you will need a good labelling. As stated in the different articles, it is very challenging to correctly label the acquired data since it is difficult to have a ground truth for GNSS signals (LOS, NLOS, multipath). To us, it seems clear that unsupervised ML will have better performance, but will require a bigger database, which is also difficult to create. It is also important to mention that during our research, we did not find any open source GNSS database. More globally, the use of ML to classify NLOS, LOS, and multipath appears to be difficult. As for the indoor/outdoor classifier [9] it cost more computational resources than the detector seen in [20] and was less precise (since the other method can detect indoors, outdoors, urban, and open sky).

### 2.6. Summary of GNSS Indicators

In this section, we have seen that many GNSS indicators that are available as the output of the receiver (C/N0, elevations, pseudo-range) can be useful in terms of context detection, but can be unreliable in certain situations. For instance, a simple threshold on C/N0 can give an idea about the context, but cannot be used as a standalone indicator. Then, we paid attention to custom indicators on the ACF function. Those indicators give much information since the ACF function provides information on the quality of the signal (NLOS, LOS, multipath), which is not affordable with simple indicators like C/N0. However, the ACF function is only available inside the receiver, and that is a huge drawback. We then reviewed the solutions that try to combine multiple indicators to see if they are more reliable than simple indicators and easier to implement than ACF ones. Most of those solutions are based on machine learning. Those techniques show good results and are able to classify LOS/NLOS without requiring access to the ACF function. However we highlighted that it is very difficult to use supervised machine learning since the labelling step is very complicated.

At this time, there is no current GNSS-based solution that can extract every context of interest in a reliable way. Another type of data is needed to perform a robust classification; that is why, in the next section, we will introduce vision-based context indicators.

## 3. Vision-Based Context Indicators

This section reviews camera-based indicators that can give information on the environmental context. The main idea is to find methods that can help or be complementary to those based on GNSS. The first part focuses on the detection of NLOS satellites thanks to sky extraction. The second part reviews scene analysis methods that can be useful to detect if we are in indoor or outdoor situations. Then, the third and fourth parts present satellite and aerial imagery that can be useful to perform a context cartography. Finally, the last part focuses on the combination of the vision-based indicators.

### 3.1. Sky Extraction

Sky extraction has been widely investigated for various applications, like meteorology [64,65], navigation [66], or even to help scene analysis [67]. Those techniques are based on different setups including visible, Ultraviolet (UV), and Infrared (IR) wavelengths, which all have their pros and cons. Far-IR reduces illumination variations, while Near IR (NIR) increases contrast between clouds and sky. UV wavelengths seem to be interesting for sky segmentation, but need to be filtered at specific peaks [68]. If sky segmentation has received so much attention, this is due to the difficulty of the task since there are many variations due to weather or time [69]. Indeed, segmenting sky into sunny or cloudy situations is totally different. Furthermore, daylight illuminance will have different characteristics than dawn or dusk illuminance (in terms of luminosity, colour temperature, shadows, etc.). Various methods exist to achieve sky segmentation, but many of them cannot be applied for our navigation purposes due to a computational time that is too high. We can cite the example of the graph cut-based solution [70] or scene parsing [71].

Sky extraction for NLOS detection in GNSS navigation has also received much attention [10,11,12,13]. Its main idea is to implement a wide angle sky-oriented camera on top of a vehicle to extract the sky part of the image. Then, satellites that appear outside the sky area when projecting their positions on the image are classified as NLOS. Those satellites are filtered so that the GNSS navigation system only uses LOS satellites in the position calculation. This considerably improves the precision of the GNSS position solution, but in certain contexts like urban canyons, only a couple of satellites are LOS; therefore, the position cannot even be computed. It is important to notice that this technology has been developed to improve the position accuracy and not to detect a context. However, based on this technology, it seems straightforward to extract some context information. As already said in the previous section, having the number of NLOS satellites can give an idea of the vehicle environment. If there are many NLOS satellites, this implies that the drone is in an urban or indoor context. If there is no NLOS satellite, we are basically in an open sky situation. From the sky extraction result, we can also create other indicators, like the presence of sky or not, the sky segment area, or the shapes of the sky boundaries. For instance, when we narrowed down the possible context to either indoors or urban canyon, the sky area indicator can be used to differentiate the two contexts. Several setups are presented below. Some are based on visible wavelengths and others on IR wavelengths. Note that there is no setup available based on UV filtering, as it is very difficult to assemble such a setup on board a vehicle.

Gakne and Petovello [11] used a camera with a standard lens (not a fisheye one) and a sensor working in the visible frequencies. The objective had an FOV (Field Of View) of 90°, which is quite small for a system operating at 360° (satellites can appear everywhere around the user position). They tried different image processing algorithms to extract the sky: Otsu, mean shift, graph cut, HMRF-EM (Hidden Markov Random Fields-Expectation-Maximization). In the end, the simplest algorithm, Otsu, proved to be the best in terms of both the processing time and the segmentation accuracy (cf. Table 4). The authors of [11] concluded that removing NLOS satellites from the position solution calculation was not a good idea since there were too few LOS satellites in many cases. However, this conclusion was due to the limited FOV of their camera and hence cannot be generalized.

**Figure 4:**
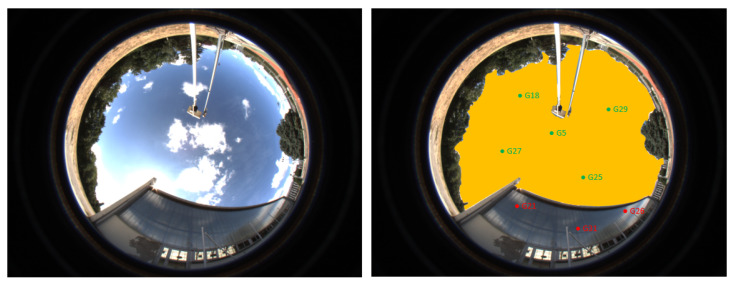
Original image (**left**) and its sky segmentation representation (**right**) with projected satellites (green = LOS, red = NLOS).

The authors of [10] used a visible camera coupled with a fisheye lens. The paper also started with a comparison of different sky extraction algorithms: colour and texture mix, mathematical morphology, k-means segmentation. The comparison was done on a database of 100 cloudy and sunny images at various locations: urban area, under trees, inside a tunnel. The mathematical morphology outperformed the other algorithms and was actually the quickest one as well (0.4 s). This algorithm started by removing the contrast parameter on each pixel of the image to pre-segment the latter. The pre-segmented image still had illuminance variations due to the sun and/or clouds. To remove these illuminance variations and obtain a homogeneous area, a geodesic reconstruction by dilatation was used. This sky segmentation algorithm reached a 93% good classification rate with a database only composed of 100 pictures of sky, but with none semi-indoor/indoor pictures where the ceiling could be classified as sky.This method is particularly efficient on high contrast images but struggles in low contrast situations which occur often in outdoor, as shown in [72]. As we not only aim to filter NLOS satellites, but use sky segmentation to help classify the context, sky segmentation must be reliable in every situation. The satellites are projected onto the image by using the STK GNSS simulation software. (Figure 4).

Their final results also showed the fact that there were less than four LOS satellites in many situations. This geodesic method of sky extraction was also used in [73]. Firstly, the NLOS satellites are removed based on the sky extraction results. Secondly, a multipaths mitigation algorithm is applied, and finally, the position solution is calculated thanks to the Vector Delay Frequency Lock Loop (VDFLL).

A complementary work to [10] was given in [74]. In this paper, they built a region classifier in four steps. The first step is to pre-process the input image with a colorimetric invariant transform. Then, the image is segmented into regions, and features are computed (essentially based on histograms) for each region. Finally, they classified regions to extract only the sky ones using a maximal similarity algorithm based on the Bhattacharyya distance. To compare the similarity, references needed to be compute beforehand. That is why two learning databases (sky and background) needed to be created. This method reached a classification rate beyond 99% on the same database as in [10] and outperformed other supervised and unsupervised classifiers (Fisher, KNN, SVM, fuzzy C-means, SRM, etc.).

**Figure 5:**
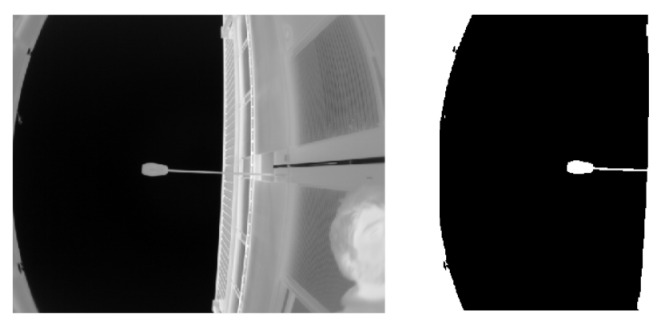
Infrared original image (**left**), sky segmentation based on Otsu threshold (**right**).

Meguro et al. [13] used another hardware setup with an omnidirectional infrared camera. This choice was firstly because an infrared camera can segment the sky very easily since it appears without any illumination variations (see Figure 5).

Infrared cameras also work at night, which is one of the limitations of visible cameras [75]. According to the authors, the omnidirectional camera was chosen since it is easier to design than a fisheye lens. One problem is that the mirrors are visible in the image. Therefore, we have a zone of uncertainty where information is missing. As done in the two other papers [10,11], they projected the satellites onto the image and concluded on the high probability of having less than four LOS satellites. However, the authors suggested two solutions to this issue. The first one is to use multi-constellation systems, using GPS and Galileo, for instance. However, when the article was written, Galileo was not fully available yet. The second solution is to filter the NLOS satellites depending on their pseudo-range residuals and their C/N0 values. Indeed, some of the satellites that are declared NLOS are sometimes just next to a sky boundary detected on the image and could be actually LOS. A similar filtering technique based on C/N0 values was also used in [40], but with different image processing. They used the Canny edge detector to create regions. Then, satellites positions were projected onto the images. The area with the highest C/N0 satellite was considered as sky, and flood filling could be started. This method does not seem robust in many situation, since it is based on edge detection and implies that the sky area is uniform (no cloud). For the same objective of NLOS confirmation, we can propose another indicator of the distance between a projected satellite and the sky, also by taking into account the uncertainties in the sky extraction, as well as in the position estimation. Wen et al. [76] proposed to weight NLOS and LOS satellites differently instead of a simple filtering of NLOS satellites. The method showed an improvement in terms of positioning, but the authors did not compare their work with other papers.

Obviously, semantic segmentation, thanks to deep learning, can also be used for sky segmentation on fisheye images. Different solutions already exist [77,78] with, respectively, 94% and 88% correct sky pixel classification. Even if using a deep learning solution on fisheye images with a training database obtained from rectilinear images is convenient (it is possible to use the LabelMe database for instance [79]), it has limited performances [80,81]. To overcome such limitations, a new training database associated with ground truth labelling with fisheye images is required. Another solution would be to modify the rectilinear image with a post-processing operation to create a fisheye-like distortion [78,80]. The main advantage of semantic segmentation is that it can be used to detect additional classes like buildings or trees, which can be beneficial for our algorithm selection purposes. There is still a major interrogation on deep learning solutions since there is no monitoring of the FPS (Frame Per Second) capability.

### 3.2. Scene Analysis/Classification

Now that we have seen the importance of what is above the vehicle (sky extraction and NLOS detection), we will take a look at what it is facing. Most of the autonomous vehicles have a frontal camera for navigation and/or mission purposes. The art of recognizing what contains a scene is called scene analysis. This is interesting for context detection, as it could help to distinguish indoors or outdoors for instance. Such decision making is referred to as scene classification. Filtering indoor scenes from outdoor ones seems like a simplification of scene classification, but in fact, it is not so simple. Indeed, the type of objects can vary greatly in both situations (plants can be found inside for instance), and external parameters such as illumination or weather can greatly influence the rendering of a picture. Different solutions exist, but there are two main families: the methods based on an analysis of the picture as a whole, which are called holistic methods, and approaches based on local descriptors, which are indicators computed on a sub-part of the image. Most of the time, the sub-parts are created by a segmentation algorithm. There are also a few methods that cannot be applied in our case. One such method is based on the labelling of the different objects of a scene to then classify it depending on the found objects (for example, if a desk, a bed, and a chair are detected in the picture, it can be classified as a bedroom). Such solutions are useful when it is necessary to precisely classify scenes to perform a mission task at a specific place. However, in our navigation application, where we only need to distinguish indoors from outdoors, the level is too high [82]. Lastly, methods based on the metadata of the picture also exist [83,84]. Indeed, with the exif (exchangeable image file) format, it is possible to have access to various parameters, which are referred as metadata. There is a wide range of possible parameters, like GPS position, time, camera manufacturer, or even information, in the picture. Those last parameters are the most interesting for image classification. Here is a non-exhaustive list of them: aperture, autofocus distance, exposure time, f-number, use of the flash, and so on. In our case, we do not have access to all the required data (for instance, it is not possible to use flash since there is no flash on a UGV setup), and therefore, we cannot use this technique.

Various local descriptors have existed in scene analysis for many years: histograms/moments (in different colour spaces: RGB [85], LUV [86,87], LST [67,88], Otha [89,90], HSV [91]), wavelets [67,85,88], Discrete Cosine Transform (DCT) [89], edge detectors (like Canny [35]), edge orientations [86,87], HOG (Histogram of Oriented Gradients), Scale Invariant Feature Transform (SIFT), etc. Most of them were reviewed in [92]. These local indicators are often combined and used as the input to classifiers. Examples of multi-feature systems with classifiers as an output were presented in [23,24,27]. The most frequent classifiers are KNN and SVM. There are also much more complex existing solutions. Some are based on brightness, gamut, exposure, and gain [28], some on a stacking of multiple indicators (in various domains), and deep learning models [29,93,94]. It is also important to note that multiple databases exist to train models (which all have their pros and cons: number of images, low resolution, image size, etc.): IITM-SCID2 [95], the fifteen scene categories [96], SUN [97], INRIA Holidays [98], Antonio Torralba’s Indoor [99,100], MIT places [101].

Holistic methods are presented as robust methods to classify scenes since deep learning solutions compare themselves to those. Holistic methods have also been compared to human recognition performances and have achieved very similar results in terms of correct classifications [102]. This result can seem very counter intuitive since the features used in those methods are global properties, which are not based on any colour or textural information, but show the potential of holistic-based technique. Two different holistic methods were presented in [25,26].

**Figure 6:**
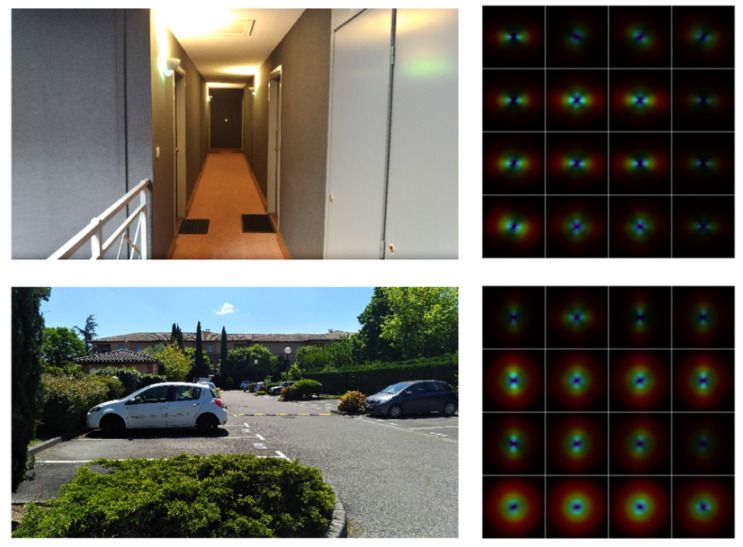
Original image and its GIST representation (indoor example on the **top**, outdoor example on the **bottom**).

The first one is called GIST. This technique is inspired by human vision and how the human brain computes information [103,104]. When a human looks at a scene, he/she will recognize the scene in less than 200 ms. This means that in a very short time period, a human can analyse a large amount of perceptual information. The goal of GIST is to create a representation in which it is easy and quick to find out what contains the scene. GIST is computed using 32 Gabor filters (with eight different orientations and four different scales). From this operation, thirty-two feature maps of the same resolution as the initial image can be obtained. Each image is divided into 16 regions using a 4 × 4 grid filter. In each of those regions, the gradient is computed (orientation and scale). Then, each of the 16 sub-regions is concatenated to compute the average value. At the end, a 512 (32 × 16)-dimensional vector is obtained and is our GIST descriptor. A representation of GIST is shown in Figure 6.

Tahir et al. [25] used a feed-forward neural network to classify their GIST vectors between indoors and outdoors. A 90.8% successful classification rate was obtained and, according to the authors, could be further improved by adding new local and global features. They also proposed to test other classifiers such as SVM to see if there was any improvement.

**Figure 7:**
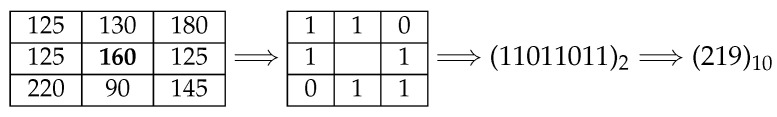
Illustration of the census transform.

The second holistic method is called CENsus TRansform hISTogram (CENTRIST) in which the authors tried to extract the structural properties of the image and remove the textural information. A first transformation called Census Transfrom (CT) is applied on every pixel of the image. The CT makes a comparison of the value of each of the eight neighbouring pixels with the value of the centre pixel. If the neighbour’s value is higher than the central value, then this neighbour pixel is set to zero, otherwise to one. Once this operation is done on the eight neighbours, a binary number is created starting from the top-left pixel to the bottom-right one (see Figure 7). This binary number is then converted to a decimal number. The centre pixel value is set to this value, which is called Census Transform value (CT value). The CT value has the advantage of being free from illumination and gamma variations. The next step is to compute the histogram of the CT values. Then, the classification is performed by the SVM and 1-NN classifiers. The last one had better results and should therefore be exploited. The classification performance of CENTRIST was compared to those of GIST and SIFT, and the result showed that CENTRIST was more reliable and faster (those comparisons were done with different datasets than the one used in the GIST article). In this comparison, the authors showed that GIST works better in outdoor situations than in indoor ones, which is not surprising since GIST was firstly developed to classify outdoor scenes [105]. Quattoni and Torralba [100] also showed the limitation of GIST to classify indoor scenes. However, CENTRIST still has some limitations. It is not rotation invariant, which is not a big problem for us since the camera and the scene are always orientated in the same direction. Besides, the colour information is totally excluded, which is surprising since it could help the scene classification greatly. Meng et al. [31] proposed an extension of CENTRIST, where the local difference magnitude is computed. The results showed a slight improvement in terms of classification.

Finally, some solutions try to mix holistic and classical approaches. Ganesan and Balasubramanian [32] used GIST in the Ohta colour space combined with CENTRIST as input features of an SVM classifier and achieved high classification performance on the IITM SCID2 and MIT datasets. Balasubramanian and Anitha [33] mixed SIFT features with enhanced GIST ones and classified the MIT-67 database with an SVM model.

In summary, scene analysis can be a good complement to GNSS signal indicators to classify the indoor from the outdoor context. Most of them are based on deep learning and present good results. Even holistic methods are now used as features for deep learning solutions. However, in the previous articles, there was no indication of the processing time of those solutions, which is one of the main limitation for navigation systems.

### 3.3. Satellite Imagery

Another way to know in which context the vehicle is evolving is to take some altitude and look at its surroundings from the sky. The goal is to create a context map of the vehicle’s environment thanks to online open-access satellite imagery databases. Much work has been done to classify the environment with remote sensing [106,107,108]. Most of the methods use the same technique, which consists of the segmentation of areas, the creation of the descriptors (shape, colour, and texture) of those areas, and finally, classification using data mining. Other solutions that detect specific attributes like buildings [109], roads [110,111], or trees [112] also exist. Satellite imagery performs well when you want to look at a large field of view at a coarse resolution.

For our objective of vehicle navigation, we need a metric of precision to distinguish such contexts as under a tree, close to a building, etc. Since satellite imagery does not fulfil this requirement, we decided to investigate aerial photography, which has a finer resolution.

### 3.4. Aerial Photography

Little work has been recently done on aerial photography since it is an alternative to satellite imagery with a much higher resolution and pixel intensities. Aerial photography actually works in a very similar manner to the satellite imagery solutions except that satellite imagery is very often hyperspectral, which is rarely the case for aerial photography. This creates major challenges to achieve a high resolution segmentation for Aerial Photography (AP) since only RGB channels are available and also some objects may have a similar aspect, but are very different semantically. For instance, tree regions are very close to grass or bush regions from a textural and colour point of view, but semantically totally different. The opposite is also true, as for example cars, which have different shapes and colours, but are semantically the same.

In the first example of [15], they tried to detect urban and wild land contexts with two different approaches: object-based and pixel-based approaches. The pixel-based algorithm uses ISODATA, an unsupervised classifier that uses the minimum spectral distance. Then, a 3 × 3 majority filter is used to find a class. As for the object-based method, the first step is to segment the image. To do so, the authors used the software called Definiens eCognition, which has a bottom-up algorithm based on spectral, textural, and user parameters. The classification is performed using a multi-scale system. The large segmented areas are classified by applying a fuzzy algorithm, while the smaller objects are classified by a KNN (K-Nearest-Neighbours) algorithm. At the end, the pixel-based method had a 62% correct classification rate, while the object-based one had 80%. The authors concluded that an object-based algorithm is more accurate than a pixel-based one for remote sensing.

Like many fields of research, AP classification can be performed using CNN, as done in [17], where AP images were classified into five classes: building, low vegetation, tree, car, impervious surface. To do so, the authors built a network where features (which were obtained by a slightly modified version of the MobileNetV2 network [113]) were shared to extract two different channels: semantic and boundary maps. The results were promising, but the main issue of this approach is that they used the NIR channel, which helps to extract grass and trees. This channel is not available in most AP databases. It has also been shown that using different scale features with fully convolutional network helps to achieve a better detection [114].

Another example of segmentation and classification of aerial images was proposed in [16]. This method is also object-based. A super-pixel algorithm (TurboPixels [115]) is applied on the image to obtain a first segmentation. Then, two features (textural and spectral) are extracted. The textural descriptor used is the LBP-HF (Local Binary Pattern Histogram Fourier) [116]. The spectral feature used is the colour histogram in the RGB domain (tests were done also in HSV, but gave poor results). Finally, two different KNN models, one for the colour and one for the texture, classify the super-pixel into a label: building, road, tree, grass, and water. The models were trained with manual segmentation, and the results were promising. Resolution can be improved with an increase in the number of super-pixels. In the article, the number of super-pixels was set to 500 without any explanation. Perhaps an increase in the super-pixels leads to more computation consumption.

It should be noted that a context map based on aerial photography can be pre-loaded on a vehicle since in most of the situations, its operation zone is known a priori. With a such solution, we will be able to have knowledge about the surrounding context if the vehicle position is estimated precisely enough with respect to the context map resolution.

### 3.5. Combination of Vision-Based Techniques

To our knowledge, there is no article in the literature that proposes to combine different vision-based techniques to extract context. This is not surprising since the sky extraction methods presented earlier are not used for the purposes of context detection, but only for NLOS detection in GNSS navigation. Furthermore, satellite imagery is used in cartography more than for context detection.

### 3.6. Summary of Vision-Based Indicators

We have seen in this section different vision-based indicators for environmental context extraction. The first part focused on different methods of sky segmentation. Methods using a colour camera and basic image processing algorithms show good results in outdoor scenarios. Semantic segmentation solutions based on deep learning also perform well. Nevertheless, there is no fisheye camera database for sky detection available, so it has to be created manually or from a rectilinear image with an additional transformation. The methods based on IR facilitate the image processing since the sky is already segmented, but the IR camera does not have the large FOV that the visible camera + fisheye lens setup has. Indeed, it is very difficult to implement such optics on an IR sensor. Although it is possible to use an omnidirectional camera as an alternative, it creates a blind spot since mirrors appear on the image. The main use of sky segmentation is satellite mitigation, by projecting a satellite’s position on the segmented image to identify LOS or NLOS. Filtering NLOS satellites improves the accuracy of the navigation solution, but also leads to the impossibility of computing a position when less than four satellites remain. Many solutions exist to solve this problem: using multiple constellations, improving NLOS detection using an association of image processing and GNSS indicators, admitting uncertainty about the projected satellite position, and so on. Sky segmentation can also be used as additional information for outdoor context detection.

Next, we focused on visual scene analysis, which can help in the outdoor/indoor classification. There are three different types of methods: local descriptors+classifier, holistic approaches+classifier, and deep learning. Local descriptors have been used for many years, but showed a lack of robustness. Hence, they were replaced by holistic methods, which consider the image as a whole to reduce variability. Currently, state-of-the-art methods combine holistic descriptors with a deep learning network. Although this achieves a high rate of correct classification, we have no information on the computation time of those techniques and if they are suitable for navigation application.

In the third and fourth part, we reviewed satellite and aerial imagery semantic segmentation with the goal to build a map of contexts. It appeared that satellite imagery resolution is too low for our navigation purposes, and hence, we focused on aerial imagery. Two different methods exist: object-based and pixel-based. The object-based algorithms have the advantage of classifying area by area, but have the drawback of requiring a pre-processing step to find each area of interest. The pixel-based techniques do not need any pre-processing, but the spatial structure object is lost, and it creates a salt and pepper effect. CNN can help to resolve this matter, but most of the proposed deep learning solutions used a database with the NIR channel, which helps to segment trees and vegetation. Therefore, we cannot confirm the classification rates of those methods.

To conclude on vision-based context indicators, the majority of works dealt with place recognition or image segmentation, but few of them were applied to the context-aware navigation.

## 4. Context Detection Based on Other Sensors

The two previous sections covered GNSS- and vision-based indicators that can be used for context detection. We are still missing indicators on the IMU, our last sensor available in the classical navigation setup. Actually, the IMU does not give interesting information on the context, as it functions in the same manner regardless of the environment. Using the IMU measurement enables detecting pedestrian steps [117], road vibrations (to check if the car is moving) [118], or how straight a vehicle is going thanks to its gyro [119]. Measurements from accelerometers and gyroscopes are also used in more complex systems to identify the behavioural context and the activity of smartphone users (for example, running, climbing stairs, inside a lift [120]), to create an activity map for example [121]. Such information will not directly help our environmental context detection. Nonetheless, using IMU data is still the best and simplest way to know if the vehicle is in motion or not. This information could be interesting for managing context detection because if the drone is idle, we have no reason to look for a new context (it is however still important to follow the evolution of the satellite constellation).

It has been shown in past years that a depth map could help the indoor/outdoor classification [30]. Generally, the depth map should be computed from a stereo vision setup, which is far from being implemented on every ground vehicle. In the previous article, the depth map has been estimated (thanks to random Markov fields) due to the lack of a stereoscopic dataset. The depth map can also be extracted thanks to LiDAR or RGB-D cameras. Papers based on those sensors are presented in the following paragraphs.

Considering LiDAR (LIght Detection And Ranging) or an RGB-D camera, it has been shown that such a sensor could be used to perform indoor/outdoor classification. The LiDAR sensor is often used on drones and other autonomous vehicles to create Digital Elevation Models (DEMs). It is usually used in addition to the standard visible camera since features cannot be found in non-textured environments. For example, in [122], they tried to detect windows using camera-LiDAR fusion. Such detection results could give us information on how far the vehicle is from windows or potentially from the exit/entrance of the buildings. Börcs et al. [123] trained a CNN model to classify buildings, vehicles, pedestrians, ground, and road clutter from a 3D point cloud, which could help us to detect outdoor scenes. Lim and Suter [18] combined features from LiDAR and a camera (heights, colours, spin image, estimated normals) to label super-voxels of a 3D point cloud as five classes (trees, trunk, building, pathway, grass) using a multi-scale conditional random field. This method could be useful for the context mapping task, but for online navigation use, it has a drawback compared to the AP method since it cannot be pre-loaded. However, it is important to note that LiDAR has not been used as a stand alone sensor for context detection so far.

RGB-D cameras, which provide colour and depth images, are also used for context detection, but only in indoor applications. Most of the time, they are used in scenes where there is a wide range of possible objects and where pixel data are not sufficient to label them. Adding another type of information with the depth map helps to label objects. Such a method is referred to as scene labelling (see [124]). Once objects have been labelled, a classifier is used to find the context depending on the objects in the scene. For instance, Gupta et al. [125] classified rooms thanks to objects detection by using an SVM classifier on the histogram of oriented gradients of the depth map. RGB-D cameras are not very useful in our case of vehicle navigation, since this technology is not reliable in outdoor environments. If any depth map data are needed for context detection, then it would be preferable to use LiDAR.

The following methods are used on smartphone devices where multiple sensors are embedded and hence are not really applicable to classical navigation systems without modifications.

Xu et al. [126] combined GNSS features (number of visible satellites, positioning error value) and light sensor data to build a decision tree with multiple thresholds. The system classified data into three families: indoor, outdoor, semi-outdoor. Both static and dynamic tests were conducted, and it appeared that in dynamic environments, the solution admitted a higher error.

References [1,127,128] proposed methods based on WiFi signals where multiple features are computed (signal-to-noise ratio, number of accessible emission points). In [1], WiFi signal measurements were combined with a GNSS-based indicator. Such multi-sensor-based context detection was also proposed in [129], where WiFi was combined this time with Bluetooth. Even more complex solutions exist such as [7,130]. Ali et al. [7] used light, internal clock, received signal strength indicator cell tower, and WiFi features in different independent modules to classify each epoch as indoors, urban outdoors, rural outdoors, and underground. Zhou et al. [130] used proximity, magnetic field, acceleration, and WiFi sensor information to detect indoor and outdoor contexts using an HMM.

Wang et al. [131] used Global System for Mobile (GSM) features to classify four different contexts: open outdoors, semi-outdoors, light indoors, and deep indoors. The authors computed the mean, standard deviation, maximum, minimum, and range for every received signals. Then, they tried machine learning algorithms to classify their data and concluded that the random forest method was the one achieving the highest score in every performance indicator (accuracy, sensitivity, precision). The big advantages of using GSM are that the signal is available basically everywhere and has very low consumption in terms of energy.

Audio can also be used to classify indoors from outdoors, as shown in [132]. The idea is to emit a sound during 100ms with variable frequencies and then capture its reverberation. Once the recording is done, Mel-Frequency Cepstral Coefficients (MFCC) are calculated and used as inputs to an SVM classifier, which is trained with labelled data. The system reached 96.26% correct classification.

Barometers can be used to detect context. For instance, they are used to determine if the setup is in motion, walking, or idle [133]. Barometers are also used for indoor navigation [134] by detecting different floors. The main advantage of the barometer is its very low consumption, but it has also drawbacks, which reduce its performances like weather/temperature changes or vibrations.

A magnetic sensor is also useful for indoor/outdoor classification. Indeed, Ashraf et al. [135] used a naive Bayes classifier with magnetic intensity features (mean, variance, kurtosis, median, standard deviation, interquartile, percentiles, squared deviation, trimmed mean, coefficient of variance, average absolute deviation) as inputs to define if the system was located inside or outside. The system achieved a classification accuracy of 83.26%.

Finally, Krumm and Hariharan [136] tried to classify indoors from outdoors by using a temperature sensor. Three different statistical distributions were computed for indoors, outdoors, and ambient temperature. Based on those distributions with ambient temperature measurements, the probabilities of being inside or outside were computed. The system had a success rate of 81%.

In this section, we reviewed different types of sensors that could be used for the environmental context detection. The diversity of those techniques shows that context detection cannot be defined precisely and that the process to find the correct classification is not deterministic. Basically, every sensor can be used since it will always add more information. It is then important to select the sensors that are more useful and reliable for the targeted application. For our navigation purposes, this paper focuses on the classical GNSS + IMU + Vision sensor setup while keeping in mind that the above listed sensors can be useful when another source of information is required.

## 5. Summary of the Different Solutions and Perspectives

This paper overviewed existing environmental context detection solutions mainly based on GNSS and vision sensors, from a navigation point of view. We firstly focused on the GNSS-based indicators and saw that C/N0 and the K-Rician factor are good indicators to detect indoor/outdoor contexts. The negative part is that a certain amount of integration time is needed to compute those values. It is also important to note that those indicators are highly sensitive and not robust to environmental variations (loss of satellites, sudden attenuation, etc.). We then took a look at the pseudo-range residual and its second derivative, which suffer from the same problems as C/N0: sensitive to noise and a need much integration time. Nonetheless, these values can be useful to distinguish LOS from NLOS satellites. Knowing the number of NLOS satellites would give information on the context: the more NLOS satellites there are, the higher the chance of being indoors (or in urban canyons) will be. Another indicator to confirm the LOS/NLOS classification (which is also important information for shadow matching) is the satellite elevation. Indeed, if the satellite is low in the sky, there is a higher chance for the signal to be blocked (NLOS). We then introduced the ACF indicators. This function is much more complicated to extract since it is only accessible inside the GNSS receiver and not as a simple output like the previous indicators. Different solutions exist, but most of them only detect multipaths without differentiating this from NLOS, which is not sufficient for context detection purposes. Finally, we presented different solutions based on multivariate GNSS data. Most of those solutions apply machine learning techniques and seem to suffer from the difficulty in creating a labelled database. Furthermore, often, the classification is just among indoors, outdoors, and intermediate, which is not fine enough for navigation adaptation purposes.

In the second part of this paper, we showed the different methods to extract context using vision information. The first method is to extract the sky segment (on an image of a wide angle camera) and to project the satellites’ positions on it in order to classify LOS/NLOS satellites. This provides us with two major pieces of information: the number of NLOS satellites and the presence of sky. Such information can give an idea about the context (indoors, urban, open sky, etc.). The sky extraction enables the GNSS navigation to improve its positioning accuracy by detecting and excluding the NLOS satellites. However, this step must be done with caution since it can lead to too few satellites available, which makes the GNSS position estimation impossible. We have seen multiple indicators that can be used in addition to help the satellite filtering like C/N0 or pseudo-range. The second vision-based solution exposed is to use a frontal camera and to analyse the scene in order to classify it and detect context. Different methods exist from local descriptors to the holistic method. Both techniques will at the end need a classifier. The third approach is to use satellite imagery, or aerial photography. We quickly saw that Satellite Imagery (SI) is not precise enough in terms of resolution for our vehicle navigation purposes, and thus, AP should be used. Most of the segmentation algorithms for AP are similar to those for SI. Those AP-based techniques are interesting since they can help us detect basically every type of context, including trees and water, that the GNSS indicators cannot (the only way to detect trees or water with GNSS is to use reflectometry, which is not possible in our case, since the receiver is near the ground level and in motion). It is also important to mention that object-based image analysis is more powerful than pixel-based (which creates a salt and pepper effect since there is no region segmentation) in the case of remote sensing, as proven in [137].

A summary table of all the reviewed methods of context detection is given in Table 5. This table is only based on the literature and not on any tests, which can explain our doubts about certain indicators (denoted as “***?***”).

## 6. Conclusions and Future Work

We showcased in this paper that current environmental context detection methods are not robust since using a single dimension descriptor is not sufficient. One of the perspectives towards robust context detection is to fuse different indicators from multiple sensors and to create a multi-dimensional context detector. To our knowledge, so far, there is no such solution in the literature trying to combine vision and GNSS indicators. This is essentially due to the fact that most of the existing works on context detection are for applications implemented on smartphones and that the use of cameras on those devices is complicated (it is not possible to have a sky-orientated camera on a smartphone for instance).

Moreover, a fair comparison between all the presented methods in this review is not possible at this time as there is no publicly available environmental context database for GNSS or visual modalities. Indeed, each work cannot be reproduced as each used its own very small dataset that was not provided to the community.

After this literature review on the context detection methods, our future work includes the development of a new method to detect environmental context consistently based primarily on vision, but with the aid of GNSS indicators for navigation adaptation purpose. As a function of the detected context, we can select the best sensor set to use or adapt to fuse measurements (for example, GNSS loose-/tight-couplings) in the navigation filter, as well as choose a more efficient algorithm to extract the information in the vision system (as shown in Figure 1). Such a context-aware adaptive navigation filter will enhance its localization accuracy and availability in a complex environment. Our new context detection method will also be applicable to make a context mapping for offline use in mission/path planning tasks.

## Figures and Tables

**Table 1:** List of the environmental contexts of interest, their impacts on GNSS/vision sensors, and the corresponding articles.

Context	GNSS		Vision		Articles
	Impact	Adaptation	Impact	Adaptation	
**Urban canyon** **(narrow street** **with tall buildings)**	Signal not available/ high positioning errors	Tight-coupling, NLOS filtering, shadow matching	None	Point- or line-based feature extraction	[8,9,10,11,12,13]
**Dense urban area** **(residential area)**	High NLOS and multipath risk	Tight-coupling, NLOS filtering	None	Classical point- or line-based feature extraction	[10,11,12,13,14,15,16,17,18]
**Low density** **urban area** **(suburban area)**	Low NLOS risk, but multipath effect possible	Doppler aiding, multipath mitigation, loose-coupling	None	Classical point- or line-based feature extraction	[10,11,12,13,14,15,16,17,18]
**Deep indoor** **(no line of sight** **to the exterior)**	Signal not available	Vision/INS coupling	Lake of texture, few robust point features	Line-based feature extraction	[1,9,16,19,20,21,22,23,24,25,26,27,28,29,30,31,32,33]
**Light indoor** **(close to door,** **window, or balcony,** **also called semi-indoor)**	Signal with high errors (due to both attenuation and reflection)	Vision/INS coupling	Lake of texture, few robust point features, glare effect	Line-based feature extraction, additional image processing step	[1,9,16,19,20,21,22,23,24,25,26,27,28,29,30,31,32,33]
**Open sky**	Perfect quality of the signal	GNSS/INS loose-coupling	None	Switch off	[1,8,10,11,12,13,15,34]
**Dense forest**	Signal attenuated and multipath	Extension of coherent integration time, Doppler aiding, multipath mitigation	Unstructured environment	Combination of points and colour features	[15,16,17,18]
**Light forest** **(couple of trees)**	Signal attenuated	Extension of coherent integration time, Doppler aiding	Unstructured environment, glare effect	Combination of points and colour features, additional image processing step	[15,16,17,18]
**Near water surface**	Tremendous number of reflections	Doppler aiding, multipath mitigation	No texture and landmarks	Switch off	[16]

**Table 2:** Carrier to Noise ratio (C/N0) ranges for different contexts (extracted from [34]).

	Outdoor	Soft-Indoor	Intermediate	Deep-Indoor
**C/N0 (dB.Hz)**	35–45	25–35	10–25	<10

**Table 3:** Performances of the different algorithms (extracted from [63]). NMEA, National Marine Electronics Association; RINEX, Receiver Independent Exchange Format.

		NMEA-Level		RINEX-Level		Correlator-Level	
		Classification Results		Classification Results		Classification Results	
		LOS	NLOS	LOS	NLOS	LOS	NLOS
Labelled	LOS	1194	98	1153	139	1279	17
Results	NLOS	286	522	288	520	179	633
F1 Score		80.42		78.11		90.39	
Overall Accuracy (%)		81.71		79.67		90.70	

**Table 4:** The performances of the different sky extraction algorithms (extracted from [11]).

	Average Processing	Accuracy (%)	
Algorithm	Time Per Image (s)	Sunny	Cloudy
Otsu	0.015	80.8	94.7
Mean shift	35.5	55.4	90.5
HMRF-EM	73.9	36.3	82.7
Graph cut	1.8	59.8	82.8

**Table 5:** List of the context indicators.

	Indicators/Context	OpenSky	LowDensityUrban	Dense Urban	Urban Canyon	Light Indoor	Deep Indoor	LightForest	Dense forest	Water
GNSS	C/N0	✔	✔	✔	✔	✔	✔	***?***	***?***	✘
	K-Rician	✔	✔	✔	✔	✔	✔	***?***	***?***	✘
	Pseudo-range	***?***	***?***	***?***	***?***	***?***	***?***	***?***	***?***	✘
	Satellite elevation	***?***	***?***	***?***	***?***	***?***	***?***	***?***	***?***	✘
Vision	Sky extraction	✔	***?***	***?***	***?***	✔	✔	***?***	***?***	✘
	No. of NLOS satellites	✔	✔	✔	✔	✔	✔	✔	✔	✘
	Aerial photography	✔	***?***	***?***	***?***	***?***	***?***	✔	✔	✔
	Scene classification	✔	✘	✘	✘	✔	✔	✘	✘	✘

(✔ = useful; ✘ = not useful; ***?*** = unclear)
